# Development of a new adapted QuinteT Recruitment Intervention (QRI-Two) for rapid application to RCTs underway with enrolment shortfalls—to identify previously hidden barriers and improve recruitment

**DOI:** 10.1186/s13063-022-06187-y

**Published:** 2022-04-04

**Authors:** Jenny L. Donovan, Marcus Jepson, Leila Rooshenas, Sangeetha Paramasivan, Nicola Mills, Daisy Elliott, Julia Wade, Domenic Reda, Jane M. Blazeby, Drew Moghanaki, E. Shelley Hwang, Louise Davies

**Affiliations:** 1 Department of Population Health Sciences, Bristol Medical School, University of Bristol, Canynge Hall, Whatley Road, Bristol, BS8 2PR UK; 2 Hines VA Cooperative Studies Program Coordinating Center and Edward Hines Jr VA Hospital, Chicago, USA; 3 Department of Radiation Oncology Greater Los Angeles VA Health Care System, UCLA Jonsson Cancer Center, Los Angeles, CA USA; 4 Department of Surgery, School of Medicine, Duke University and Duke Comprehensive Cancer Center, Durham, NC USA; 5 VA Outcomes Group, White River Junction, VT USA; 6 Geisel School of Medicine at Dartmouth and Department of Surgery (Otolaryngology Head & Neck Surgery) and The Dartmouth Institute for Health Policy and Clinical Practice, Hanover, NH USA

**Keywords:** Randomised trials, Recruitment, Enrolment, Recruitment intervention, Recruitment shortfall, Remedial strategies, QuinteT Recruitment Intervention, Accrual

## Abstract

**Background:**

Many randomised controlled trials (RCTs) struggle to recruit, despite valiant efforts. The QRI (QuinteT Recruitment Intervention) uses innovative research methods to optimise recruitment by revealing previously hidden barriers related to the perceptions and experiences of recruiters and patients, and targeting remedial actions. It was designed to be integrated with RCTs anticipating difficulties at the outset. A new version of the intervention (QRI-Two) was developed for RCTs already underway with enrolment shortfalls.

**Methods:**

QRIs in 12 RCTs with enrolment shortfalls during 2007–2017 were reviewed to document which of the research methods used could be rapidly applied to successfully identify recruitment barriers. These methods were then included in the new streamlined QRI-Two intervention which was applied in 20 RCTs in the USA and Europe during 2018–2019. The feasibility of the QRI-Two was investigated, recruitment barriers and proposed remedial actions were documented, and the QRI-Two protocol was finalised.

**Results:**

The review of QRIs from 2007 to 2017 showed that previously unrecognised recruitment barriers could be identified but data collection for the full QRI required time and resources usually unavailable to ongoing RCTs. The streamlined QRI-Two focussed on analysis of screening/accrual data and RCT documents (protocol, patient-information), with discussion of newly diagnosed barriers and potential remedial actions in a workshop with the RCT team. Four RCTs confirmed the feasibility of the rapid application of the QRI-Two. When the QRI-Two was applied to 14 RCTs underway with enrolment shortfalls, an array of previously unknown/underestimated recruitment barriers related to issues such as equipoise, intervention preferences, or study presentation was identified, with new insights into losses of eligible patients along the recruitment pathway. The QRI-Two workshop enabled discussion of the newly diagnosed barriers and potential remedial actions to improve recruitment in collaboration with the RCT team. As expected, the QRI-Two performed less well in six RCTs at the start-up stage before commencing enrolment.

**Conclusions:**

The QRI-Two can be applied rapidly, diagnose previously unrecognised recruitment barriers, and suggest remedial actions in RCTs underway with enrolment shortfalls, providing opportunities for RCT teams to develop targeted actions to improve recruitment. The effectiveness of the QRI-Two in improving recruitment requires further evaluation.

**Supplementary Information:**

The online version contains supplementary material available at 10.1186/s13063-022-06187-y.

## Background

Well-conducted pragmatic randomised controlled trials (RCTs) are needed to provide high-quality evidence to ensure optimal patient outcomes and efficient use of resources. Tools are available to support trial design [1] or access evidence [2], and strategic initiatives aim to improve conduct [3, 4], but completing RCTs within targets and budgets remains problematic [5–7]. Only around one half of RCTs meet their enrolment target [6]. Failures with recruitment have been identified as the biggest threat to the completion of RCTs, and poor recruitment can compromise statistical power, delay acquisition of findings, and lead to costly extensions or premature closure [8, 9]. Only a small number of simple interventions such as incentives and follow-up messages have robust quantitative evidence of effectiveness [10, 11]. Meanwhile, systematic reviews of qualitative research have called for interventions that better reflect the complexity of the enrolment process, including the communication strategies of recruiters and the perspectives of patient participants [12, 13].

The QuinteT (Qualitative research integrated in Trials) Recruitment Intervention (QRI) is one such complex intervention [14]. It was first developed during recruitment to the UK ProtecT (Prostate cancer testing and Treatment) trial comparing active monitoring, surgery, and radiotherapy for localised prostate cancer [15, 16]. This initial approach was refined through a synthesis of evidence combining ProtecT and QRIs in five other similarly challenging RCTs, which also elucidated the fragile, protracted, and evolving nature of the recruitment process [17, 18]. The QRI protocol was published in 2016 to explain the design, theoretical underpinnings, and methods [14] and to facilitate its use by other groups [19]. The QRI aims to optimise recruitment in RCTs tackling important healthcare questions and anticipating recruitment challenges because of controversy, divergent clinical opinions, or inclusion of very different interventions (such as surgery v. radiation or invasive v. conservative/de-escalation options). A QRI involves close integrated working with the RCT team from the design stage onwards, aiming to prevent the development of anticipated barriers, and then prospectively identifying and understanding the challenges that arise along the recruitment pathway from patient screening to randomisation. Remedial actions are then targeted to optimise recruitment and ensure timely completion of accrual.

The QRI is a two-phase intervention [14] (Table 1). During phase I, a researcher collects and analyses data to understand the RCT’s recruitment processes and barriers prospectively through:

(i) Interviews with key RCT personnel (triallists and recruiters), and sometimes patients—to understand the planned RCT design and recruitment intentions and experiences
(ii) Mapping a patient’s pathway through the recruitment process, using screening logs and analysis of screening/accrual monitoring data—to understand organisational issues arising from the implementation of the protocol in clinical sites
(iii) Audio-recording of consultations/appointments where the RCT is presented by recruitment staff to eligible patients and consent is sought for participation—to understand how the study is presented to patients and how they react to it
(iv) Content analysis of study documentation including the RCT protocol and patient information/consent form—to understand the details that need to be actioned by sites and how well the patient-facing study materials reflect the RCT design.

**Table 1:** Aims and research methods of the QRI protocol [14] and aspects included in the QRI-Two

QRI phase I	Aims	Analysis method
i) Interviews with RCT staff (investigators and front-line recruiters); sometimes patients	To explore key aspects of RCT design and recruitment pathway, views about interventions, intentions in relation to recruitment.	Qualitative thematic analysis
ii) Mapping a patient’s pathway through screening, eligibility, and randomisation, using screening logs and flow charts; accrual data	To identify patterns in screening and eligibility assessment and accrual data, for example differences in numbers screened, eligible, approached, and randomised in sites	Qualitative content analysis and simple quantification
iii) Audio-recordings of recruitment appointments where the RCT is presented by recruiters to eligible patients	To assess clarity of study presentation by recruiters (compared with intentions in interviews) and reactions of patients to study terminology	Qualitative thematic and conversation analysis
iv) Scrutiny of study documents, e.g. RCT protocol, patient information/consent forms, study website^**a**^	To understand RCT design, purpose, evidence base, inclusion/exclusion criteria, recruitment pathway, and interventions in protocol, and clarity and consistency with patient information.	Documentary content analysis
**QRI phase II**	**Aims**	
a) Present phase I findings to the CI/RCT team^**b**^	To summarise details and evidence from the phase I findings about clear obstacles and hidden challenges to recruitment and propose remedial actions	
b) Develop the plan of actions to optimise recruitment, and implement jointly with RCT team	To agree actions to optimise recruitment based on evidence from (a), e.g. feedback and training, site reviews, clarification of eligibility criteria, collection of additional accrual data, changes to study information, and repeat data collection, analysis, and reporting iteratively as above, as required	

*Aspects of QRI protocol included in QRI-Two*:

^a^Data collection method included in QRI-Two

^b^Presentation of QRI-Two findings specifically in a workshop format to facilitate discussion of newly diagnosed recruitment barriers and potential remedial actions

The analysis of these data from phase I of a QRI finds two types of recruitment barriers: ‘clear obstacles’ (anticipated, often organisational issues known to the RCT team) and ‘hidden challenges’ (difficulties relating to underlying aspects of the RCT design that cause discomfort for recruitment staff and/or patients, and that are often unknown to—or underestimated by—RCT teams) [17]. Each RCT will have its own array of clear obstacles (such as clinic space and time, staff availability) and hidden challenges (such as variable clinician equipoise, strong patient preferences) that interact to hamper recruitment. Phase I of the QRI aims to understand and provide evidence to reveal how these obstacles and challenges impede recruitment so that they can then be discussed with the RCT team and a collaborative plan of remedial actions to tackle the barriers can then be developed and implemented during phase II [14]. Phase II actions to improve recruitment are tailored to the RCT’s particular difficulties and may include, for example, additional support and training for recruiters in relation to equipoise and presentation of the RCT, increased collection of accrual monitoring data, and unpacking terminology to improve the clarity of patient information [20–23].

An observational study of five completed QRIs found evidence of significantly increased recruitment rates in three RCTs and study completion to time and target in all five RCTs [24]. RCTs in oncology and surgery with invasive versus monitoring or placebo arms, or comparing surgical procedures (all with very difficult recruitment issues), have completed recruitment with support from QRIs and published outcomes [16, 25–27]. Many other RCTs with QRIs have completed recruitment and are in follow-up, or continuing to enrol. A small number ceased recruitment, with evidence explaining their insurmountable difficulties [28, 29].

The QRI was originally designed to be integrated with RCTs anticipating recruitment problems so there would be time to collect and analyse data to identify and understand the recruitment barriers (phase I), and then implement actions to optimise recruitment in phase II. However, very many RCTs launch with targets to complete accrual but then encounter enrolment shortfalls, some serious enough to threaten premature closure, even with valiant efforts by the RCT team [7–9]. The QRI protocol was applied to some RCTs underway with enrolment difficulties when requested, but there was mixed success, primarily because of the time needed to obtain governance approvals and undertake data collection and analysis, particularly when these RCTs already needed urgent remediation [30]. The QRI required adaptations to provide more rapid insights about recruitment barriers operating in ongoing RCTs, particularly the existence and manifestation of hidden challenges [17]. This paper presents the development, application, and finalisation of the QRI-Two intervention—designed to provide a rapid ‘diagnosis’ of recruitment barriers and proposed remedial strategies to improve recruitment in ongoing RCTs with enrolment shortfalls.

## Methods

The aim of this study was to investigate how to adapt the published QRI to better fit the needs of RCTs already underway with enrolment shortfalls. The objectives were to:

- Identify methods of data collection and analysis from phase I of the QRI protocol [14] that could be rapidly applied to ongoing RCTs and any new methods needed to develop the new intervention (QRI-Two). This was undertaken through a retrospective review of data collected in all QRIs applied to ongoing RCTs with enrolment shortfalls between 2007 and 2017—see ‘(a) Review to identify optimal methods of data collection and analysis for the QRI-Two’.
- Apply the adapted QRI-Two intervention to consecutive ongoing RCTs and investigate its feasibility and the recruitment barriers and strategies for improvement identified. This was achieved through the application of the QRI-Two in all ongoing RCTs from 2018 to 2019—see ‘(b) Application of the QRI-Two to ongoing RCTs’.
- Produce a finalised QRI-Two protocol for future use—see ‘(c) Finalising the QRI-Two protocol’.

**Fig. 1:**
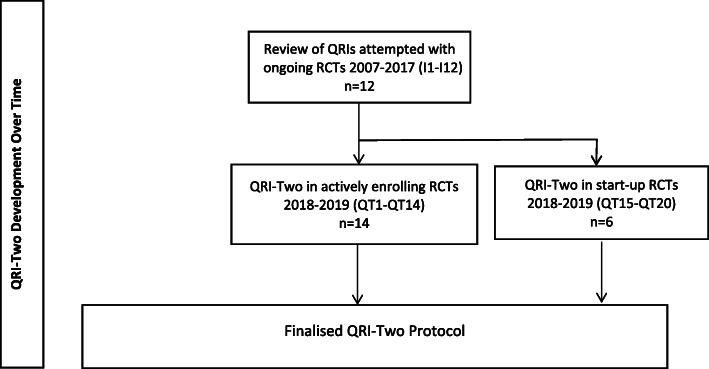
Study schema showing QRI-Two development over time (2007–2019)

The study design is illustrated in Fig. 1. A CReDECI 2 checklist is provided (Table 2) outlining the process of development and evaluation of this complex intervention [31].

**Table 2:** CReDECI 2 [31] checklist for QRI-Two

Item	Description	Page
**First stage: Development**		
1 Description of the intervention’s underlying theoretical basis	Theoretical basis of QRI-Two from two underlying theories: a) RCTs are Complex Adaptive Systems and local sites and recruiters implement the RCT protocol with some freedom, necessitating understanding of contextual factors influencing behaviour [32] b) QRI methods identify known ‘clear obstacles’ and unknown or underestimated ‘hidden challenges’ to recruitment that can be revealed and understood in phase I, and then addressed in phase II through targeted remedial actions [17]	3
2 Description of all intervention components, including the reasons for their selection as well as their aims / essential functions	QRI-Two phase I comprises: Analysis of RCT documents and accrual data to identify barriers to recruitment, especially hidden challenges revealed by QRI-analysis; plus workshop to present diagnosis of recruitment barriers and discuss remedial actions	9, 10
3 Illustration of any intended interactions between components	Workshop facilitates discussion of recruitment barriers in the context of the RCT team’s clinical knowledge and enrolment experiences	10
4 Description and consideration of the context’s characteristics	Each RCT’s documents and accrual data provide context. The QRI researcher draws on knowledge and experience of other QRIs	10
**Second stage: Feasibility and piloting**		
5 Description of the pilot test and its impact on the definitive intervention	Four RCTs confirmed the feasibility of undertaking the streamlined process of analysis rapidly and the value of the workshop discussion with the RCT team.	7
**Third stage: Evaluation**		
6 Description of the control (comparator) and reasons for the selection	Six RCTs at the start-up stage (control) were compared with RCTs underway with enrolment shortfalls. This clarified that the QRI-Two had greater relevance for actively-enrolling RCTs with accrual data.	7
7 Description of the strategy for delivering the intervention within the study context	QRI-Two is delivered by two QRI researchers. The analysis and workshop can be undertaken within 2-6 weeks of receipt of RCT documents and accrual data.	10
8 Description of all materials or tools used to deliver the intervention	RCT team need to supply RCT documents (protocol and patient-information materials) and all available accrual data. QRI researchers undertake analysis and present findings for discussion at workshop.	10
9 Description of fidelity of the delivery process compared with the study protocol	All RCTs provided the documents and accrual data as required. The QRI-Two analysis and workshop were delivered according to protocol in all cases.	7
10 Description of a process evaluation and its underlying theoretical basis	The feasibility of the QRI-Two was assessed in terms of undertaking the analysis and workshop rapidly, recruitment barriers identified, remedial actions proposed, QRI-informed training delivered, and feedback from RCT teams about the usefulness of the QRI-Two.	6, 7
11 Description of internal facilitators and barriers potentially influencing the delivery of the intervention	Scheduling the workshop and including the most appropriate members of the RCT team was sometimes difficult because of clinical or other commitments.	
12 Description of external conditions or factors occurring during the study which might have influenced the delivery of the intervention or how it works	The variable quantity and quality of accrual data provided by the RCT teams meant a greater reliance on clues about the existence of recruitment barriers rather than evidence in some RCTs.	11
13 Description of costs or required resources for intervention delivery	QRI-Two analysis and workshop requires time-commitment of several days for senior researchers. The cost is a fee to cover this time, depending on scale and complexity of RCT.	

### (a) Review to identify optimal methods of data collection and analysis for the QRI-Two

All data collected in QRIs applied to ongoing RCTs with enrolment shortfalls between 2007 and 2017 were documented and reviewed to investigate which aspects of the QRI protocol (data collection and analysis—i to iv above) could be completed and whether new methods were required to fit the needs of ongoing RCTs. Information was collected about the characteristics of the RCTs, how long it took to complete data collection and analysis, the recruitment barriers that were identified, remedial actions that were proposed, the level of funding support provided for the QRI, and recruitment completion or trial closure. Data were summarised in a table (Table S1) and the descriptive findings were used to develop the QRI-Two. The final four QRIs acted as a pilot to investigate its rapid application to RCTs underway with enrolment shortfalls.

### (b) Application of the QRI-Two to ongoing RCTs

The newly developed QRI-Two was then applied to all ongoing RCTs with recruitment shortfalls collaborating with the QuinteT team between 2018 and 2019. Data were collected prospectively about the details of the RCTs and the findings from the analysis of RCT documents (protocol and patient information materials) and screening/accrual data. Recruitment barriers were identified using previously published QRI methods which use intensive comparison and triangulation of all available sources of qualitative and quantitative data to identify the recruitment difficulties affecting each RCT [33].

RCT teams were asked to describe recruitment barriers they were aware of. The QRI-analysis sought to provide new insights about those barriers and also to identify ‘hidden challenges’—those previously unknown to the RCT teams or whose impact had been underestimated [17]. Also documented were the remedial actions proposed to improve recruitment discussed during the workshop. These data were collected by the researcher leading the QRI-Two and then compiled and anonymised in tables to facilitate comparison and synthesis by JLD, MJ, and LR (Tables S2–3). The feasibility of the application of the QRI-Two protocol was assessed according to the ability to apply it in full, recruitment barriers identified, remedial actions proposed, and informal feedback received from RCT teams.

### (c) Finalising the QRI-Two protocol

The QRI-Two protocol for future use in actively enrolling RCTs with recruitment difficulties was finalised based on the optimal methods identified in (a) and the findings from its application to RCTs in (b) in terms of feasibility and acceptability to RCT teams.

## Results

The findings of this study include data from all forms of QRI or QRI-Two undertaken with ongoing RCTs between 2007 and 2019 (*n*=32 RCTs). A QRI or QRI-Two began after a direct request for help from the RCT team or a referral from a funding/oversight body because of concerns about serious recruitment difficulties, enrolment shortfalls, or imminent closure. Funding provided for QRI-Two work varied, ranging from expenses only to funding of researcher posts. In the sections below, the development, application, and finalisation of the QRI-Two are presented (see Fig. 1 for study outline).

### (a) Review to identify optimal methods of data collection and analysis for the QRI-Two

During 2007 to 2017, 12 RCT teams requested a QRI because their trials were underway with severe recruitment difficulties or threatened with closure. All were parallel group RCTs—three in oncology, seven surgery, and two medicine. Nine compared an invasive procedure with a conservative intervention. Data from these 12 RCTs were reviewed retrospectively and tabulated (Table S1). The review found that it was only possible to complete the four elements of QRI protocol data collection (interviews, recruitment pathway mapping, audio-recordings of appointments, and content analysis of study documents—Table 1) in only four of these 12 RCTs (I3, I5, I7, I8). These four RCTs had time and funding to allow RCT protocol modifications and governance permissions for new data collection and analysis; the remainder had insufficient time for these activities and minimal or no funding. The QRI protocol methods applied most frequently were the qualitative content analysis of RCT documents (in 11 RCTs) and analysis of accrual screening/monitoring data overall and by site (8 RCTs).

The 12 RCT teams reported they had tried everything they could think of to tackle recruitment pathway and organisational issues they had anticipated, and that despite this, enrolment remained problematic. The review revealed that the QRI analyses were able to identify several other recruitment barriers in all 12 RCTs (Table S1). Previously unrecognised (hidden) challenges newly identified included discomfort with equipoise (11 RCTs), unbalanced study presentation and/or unclear terminology (9 RCTs), variations in site assessments of patient eligibility (8 RCTs), difficulty approaching patients (6 RCTs), and strong patient preferences for particular interventions (3 RCTs). There were also new insights about known organisational issues related to recruitment pathways in 7 RCTs. Illustrative examples of how these newly identified (previously hidden) challenges were found in three RCTs are given in Table 3.

**Table 3:** Illustrative examples of QRI analysis from three of the initial 12 RCTs showing how the evidence/clues found in the RCT’s documents and accrual data supported the identification of new previously hidden/underestimated recruitment barriers and suggested remedial actions

RCT	Interventions	QRI findings	Challenges identified	Suggested QRI remedial actions
I2	Three drugs for feverish infants (primary care/ paediatrics)	Accrual data showed only a small percentage of eligible parents were enrolled [21]. Interviews revealed that recruiters were uncomfortable discussing the RCT with parents whose children they considered too unwell [18]. Recruiters approached only a small number of parents—those whose children were not distressed and whom they believed would agree to participate [34].	Issues with eligibility, equipoise, approaching patients	Reconsider interpretation of eligibility criteria with RCT team [17]. Provide QRI training/support for recruiters about how to approach all eligible parents [35].
I5	Radiation v. surgery for cancer	Accrual data showed wide variations between sites in numbers of RCT-eligible patients [21]. In interviews, specialists admitted they were uncomfortable introducing the RCT to patients with particular disease features, even though they met the RCT eligibility criteria [18]. Recruiters reported that patients declined randomisation because they preferred one of the interventions (usually surgery). Analysis of patient information found terminology describing surgery as the ‘gold standard’ and a lack of balance in descriptions of the interventions [28].	Issues with eligibility, equipoise, patient preferences, study presentation	Discuss interpretation of eligibility criteria and equipoise with RCT site PIs [20]. Provide QRI training/support for recruiters about how to manage patient preferences [22]. Provide suggestions to change patient information [23].
I4	Social (employment) intervention v. usual care, psychiatry	Interviews, audio-recordings, and analysis of patient information revealed that recruiters favoured the intervention arm over the control [18]. Recruiters found aspects of the randomised design difficult and misrepresented it [23]. Recruiters approached only patients they considered well enough for the RCT’s social intervention.	Issues with equipoise, study presentation, approaching patients, eligibility	Discuss equipoise issues and definition of usual care with RCT team [20]. Provide training/support for recruiters about RCT design and how to approach all eligible patients [35].

A *new initiative* (not included in the published QRI) that was used in many of these QRIs was to present the findings of the analysis to the RCT team in a workshop format to enable open discussion of the evidence and clues that supported the identification of new ‘hidden’ barriers found in the RCT’s documents and data (examples in Table 3). Discussions were often vigorous as RCT team members considered the relevance of these newly identified, previously hidden challenges to their RCT. RCT teams were provided with a copy of the workshop presentation and suggestions for remedial actions. Included in the workshop were some elements of QRI-informed training for recruiters (11 RCTs) and ‘tip-sheets’ to support recruiters with study presentation (6 RCTs). One RCT (I7—comparing surgery, placebo, and no intervention in orthopaedics) provided data for a formal evaluation. This observational before-and-after evaluation found strong evidence that the QRI increased recruitment (OR 2.66 (95% CI 1.90–3.72) [24] and this RCT completed recruitment and published its outcomes [25]. Six other RCTs subsequently completed recruitment and acknowledged contributions from the QRI: I2, I3, I4, I6, I7, and I9 (Table S1). Two RCTs closed (I5, I10), in part because of QRI evidence about their insurmountable difficulties (e.g. [28]). The other three continue to recruit to date (Table S1).

These findings showed that even with limited application of QRI methods, recruitment challenges previously unknown/underestimated by RCT teams could be found, remedial actions could be proposed, and in some cases were implemented successfully. Further scrutiny revealed that the protocol stabilised in the final four RCTs (I9–I12) with reliance on a detailed analysis of RCT documents and accrual data, and presentation of the findings in workshop-style meetings. These four studies were essentially a pilot of the refined QRI-Two intervention.

### (b) Application of the QRI-Two to ongoing RCTs 2018–2019

#### QRI-Two protocol

The findings above indicated that the QRI-Two protocol for application to ongoing RCTs in 2018–2019 should include:

- QRI-informed analysis to diagnose the RCT’s recruitment difficulties.
  To include a rapid content analysis of existing RCT documents (patient information materials, consent forms, RCT protocol), and analysis of all available screening/eligibility and accrual monitoring data, alongside the RCT team’s summary account of recruitment difficulties.
- Workshop (c.2–2.5 h) led by the QRI team and attended by the chief/principal investigator and key RCT team members of their choice (usually including the statistician, trial manager/coordinator, data coordinator, and clinical investigators) to discuss the findings from the analysis.
  To include a detailed presentation of the findings from the QRI-Two analysis, plus relevant short QRI-informed training sessions, and detailed discussion of potential remedial actions that could improve recruitment in the context of the RCT team’s experiences and knowledge.

A key issue to be investigated was whether these methods on their own could identify previously hidden challenges to recruitment—without the other items in the QRI protocol (see Table 1).

#### Application of the QRI-Two, 2018–2019

##### RCT details

During 2018 and 2019, the QRI-Two was applied in 20 RCTs. At the outset, funding or collaboration was put in place only for applying the QRI-Two as describe above. Ten RCTs were in the USA, seven in the Netherlands, and three in the UK. There were 13 of parallel group design, six placebo-controlled, and one factorial; 15 had two-arms (five three arms); nine at least one drug arm and 11 at least one surgery arm; seven were surgery v. conservative option, two surgery v. radiation, and two compared surgical procedures. Six RCTs were in orthopaedics, three cardiovascular medicine/surgery, and 11 other specialties (Tables S2 and S3). Of these 20 RCTs, 14 were actively recruiting and had evidence of severe enrolment shortfalls (Table S2). Although our intention was only to test the QRI-Two in actively enrolling RCTs, the two oversight bodies requested it should also be applied to six RCTs about to launch because they were anticipating severe enrolment difficulties (Table S3). All RCTs were included in the analysis for completeness.

##### Feasibility

The rapid analysis of RCT documents/data and workshop presentation were completed with all 20 RCTs. However, in the six RCTs at the launch/start-up stage, the lack of enrolment (and thus accrual data) meant the QRI-Two analysis could focus only on identifying potential/hypothetical issues. Feedback was positive from some RCTs (Table S3), but discussion in these pre-recruitment RCTs was limited without enrolment experience and this confirmed that the QRI-Two was most relevant for RCTs underway with enrolment shortfalls (as intended and designed).

In contrast, there were highly engaged workshop discussions with all actively enrolling RCT teams and very positive feedback (Table S2). Discussions were highly engaged and often intense as RCT team members considered the veracity of the newly identified recruitment barriers in relation to their experience of enrolment in their RCT.

The remaining findings focus on the 14 actively enrolling RCTs—for which the QRI-Two was designed.

##### Recruitment barriers identified in 14 actively enrolling RCTs

In their summaries of their recruitment difficulties, all RCT teams listed organisational issues such as staff availability and a shortage of eligible patients as barriers and some mentioned they thought specialists might not to be in equipoise. All RCT teams described many improvement strategies they had implemented, including additional site training, incentives, and direct calls/visits to sites. However, all indicated that recruitment problems persisted and for many they seemed insurmountable and thus they were unsure what else they could do to improve enrolment.

The QRI-Two analysis identified new recruitment issues or new insights into known barriers in all 14 actively enrolling RCTs (Table S2), with between three and six previously unknown or underestimated (hidden) challenges diagnosed in each RCT (Table 4). The most common challenges identified were study presentation issues (14 RCTs), discomfort with equipoise (13 RCTs), and variations in assessments of eligibility criteria (12). These were identified as follows:

**Table 4:** Recruitment challenges identified in the QRI-Two with 14 actively recruiting RCTs

RCT (no. of challenges)	Recruitment challenges found						
	Unclear study presentation	Issues with equipoise	Limited accrual data/analysis	Issues with eligibility	Complex recruitment pathway	Patients with strong preferences	Issues with approaching patients
QT1 (6)	x	x	x	x	x	x	
QT2 (5)	x	x	x		x	x	
QT3 (5)	x	x	x	x		x	
QT4 (4)	x	x	x	x			
QT5 (4)	x	x	x	x			
QT6 (4)	x	x	x	x			
QT7 (5)	x	x	x	x		x	
QT8 (3)	x	x		x			
QT9 (6)	x	x	x	x	x		x
QT10 (4)	x		x		x		x
QT11 (5)	x	x	x	x	x		
QT12 (6)	x	x	x	x	x	x	
QT13 (6)	x	x	x	x	x		x
QT14 (5)	x	x	x	x	x		
Total number of RCTs	14	13	13	12	8	5	3

*Study presentation issues*: None of the RCT teams had previously considered this as a potential source of recruitment difficulties. Most had not reviewed study materials since gaining governance approvals at the start of the RCT. Evidence and clues for this hidden challenge included descriptions or terminology in RCT materials of one intervention being more detailed and positive than another, or a lack of clarity about why the RCT was needed, or what the ‘control’ intervention comprised. Other issues included a lack of information about randomisation or describing it only as ‘like the toss of a coin’, which has been shown to be difficult for patients [23]. Most teams wanted to revise study materials after the QRI-Two workshop.

*Issues with eligibility and equipoise*: In 12 RCTs, these issues were intertwined. There were examples of RCT protocol inclusion/exclusion criteria that were imprecise, such as ‘other’, or requiring non-clinical judgements such as ‘suitable’, meaning that these were being interpreted variably. In some RCTs, there were inconsistencies in descriptions of eligibility criteria or interventions between the protocol and patient information. QRI-analysis of the accrual data found wide variations in numbers of patients screened by sites, or in numbers excluded according to particular criteria. Some sites found reasonable numbers of eligible patients but then reported very low numbers approached about the RCT or randomised. When the findings were compared with sections in the RCT protocol or patient information, patterns associated with discomfort with eligibility and equipoise found in other QRIs were exposed [18, 20].

Other previously hidden challenges included complex *recruitment pathways* (8 RCTs), strong *patient preferences* (5 RCTs), and difficulty *approaching patients* (3 RCTs). While there was evidence of the top three challenges in most RCTs, few RCTs had the same pattern of issues, suggesting the QRI-Two found an array of hidden challenges unique to each RCT (Table 4).

Another previously hidden issue identified in most RCTs (13) was the limited collection or analysis of accrual data by the RCT team. Some RCTs had collected basic eligibility or screening data but usually analysed this only to calculate accrual progress against original overall or site level targets. Where site level data on screening or eligibility criteria were available, the QRI-researcher demonstrated how analysis of variations could provide clues about organisational barriers and where potentially eligible patients were being lost from the accrual process using the QRI-SEAR framework [21]. Where data were not available from the RCT team, anonymised data from other RCTs were shown to illustrate the value of such information. Teams indicated they found this very helpful (Table S2).

##### Workshop

The presentation of the QRI-Two analysis findings in a workshop format provided a forum for intense debate about the newly diagnosed recruitment barriers in the context of the RCT team’s experiences of recruitment and knowledge about the clinical setting of the RCT. Short QRI-informed training sessions using material from other QRIs were also provided to enable further understanding of the hidden challenges (Table S2). The workshop and training sessions concluded with a focus on potential remedial actions to improve recruitment based on the newly diagnosed barriers.

##### Further collaboration

Although further collaboration or evaluation was not planned as part of the QRI-Two commissioning process, four RCTs requested additional help after the workshop. QT7 and QT8 requested and received additional QRI-informed training to assist with the development of their own remedial strategies. QT1 and QT2 supported further longer-term collaboration—akin to phase II of the published QRI [14]—including the development and implementation of a joint remedial action plan:

In QT1, this was undertaken over 12 months as part of a visit to the USA by JLD, during which processes for regularly monitoring and analysing accrual data were put in place, QRI-informed training was provided at study meetings, site review visits/calls were organised, study-wide ‘tip’ sheets were provided for recruiters, and patient-facing information materials were revised. While formal evaluation was not undertaken, the QT1 chief investigator reported ‘we have hit our ramp-up goal almost two months earlier than our projected date… Your assistance and intervention was very instrumental in helping achieve this goal’. Collaboration then paused because of the end of the visit and suspension of QT1 during the Covid-19 pandemic. It re-started in 2021 having doubled the number of recruiting sites.

In QT2, findings from the QRI-analysis contributed to an extension to continue recruitment, and so collaboration continued through LD based in the USA supported by JLD in the UK. Implementation of the joint action plan was delayed by the need to revise the RCT protocol, work with the external data team to develop processes for producing overall and site-level accrual data, and several lengthy rounds of governance review procedures to revise the consent form and patient-facing information materials. QRI-informed training, tip-sheets, and site visits/calls commenced with the new study materials in October 2020, and an evaluation is planned in 2022. In the meantime, the principal investigators of QT2 stated, ‘we are all now resolute that QuinteT holds the solutions we need to improve the quality of what we do in the RCT’, and ‘this has been a tremendous effort and is an exemplar of what should be provided for every trial’.

In one RCT, the oversight body found the QRI-Two findings helpful in providing evidence to confirm the RCT team’s view that the recruitment difficulties were insurmountable, and the RCT was closed. According to RCT registration websites accessed in August 2021, six of the remaining RCTs continue to recruit, two were paused (Covid-19), and one was in follow-up.

### (c) Finalising the QRI-Two protocol

Findings from (a) and (b) above were reviewed to finalise the QRI-Two protocol for future use (Table 5). An important finding was that the QRI-Two approach is best applied to RCTs already underway with enrolment shortfalls. This was the original intention, but the inclusion of six RCTs at the start-up stage (at the request of oversight bodies) helped to confirm that enrolment data and RCT team experiences are needed to diagnose actual (rather than only anticipated) recruitment challenges. Another important confirmation was that the QRI-analysis was able to successfully identify an array of previously hidden challenges in each of the 14 actively enrolling RCTs. Hidden challenges are (by definition) difficult to find and the QRI-Two analysis revealed that these were discovered by searching for the following evidence or clues, as follows:

**Table 5:** Finalised QRI-Two protocol

**QRI-Two protocol**	
Analysis to diagnose the RCT’s recruitment barriers	Analysis of: Existing RCT documents (RCT protocol and patient information materials) to assess clarity of RCT design and details and study presentation RCT team’s summary of recruitment difficulties to understand known barriers Accrual monitoring data to assess complexity of recruitment pathway and variations in eligibility assessment and recruitment overall and by site
Workshop (2–2.5 h) with RCT team to discuss findings of QRI-Two analysis and potential remedial actions	Workshop facilitated to: Present the findings from the QRI-Two analysis and relevant short QRI-informed training sessions Discuss findings in the context of the RCT team’s enrolment experiences Consider and propose potential remedial actions to improve recruitment
**Further work needed to tackle newly-diagnosed recruitment barriers**	
RCT team to decide whether recruitment barriers are insurmountable, or can be tackled with remedial actions to improve enrolment	
Development and implementation of remedial actions to improve recruitment	(a) RCT team to devise own plan and remedial strategies, or (b) Support further collaboration with other groups* *Could include phase II of the QRI [14]

*RCT protocol*: Complicated, inconsistent, or unclear descriptions of underpinning evidence, RCT rationale/design, clinical/recruitment pathways, inclusion/exclusion criteria, and details of interventions can indicate hidden issues with equipoise, study organisation, and study presentation.

*Patient information*: Unclear descriptions of the aims of the RCT, inconsistent or imbalanced descriptions of interventions, or obtuse terminology can indicate problems with study presentation, clinician or patient equipoise, and intervention preferences.

*Screening/eligibility and accrual monitoring data*: Data variations by site that are usually ascribed to site size or patient case-mix can provide indications of discomfort with eligibility criteria, equipoise, willingness to approach patients about the RCT, the presence of patient preferences, and the quality of patient information.

The triangulation of the evidence and clues [33] enabled the diagnosis of specific challenges to be presented in the workshop. The implementation also confirmed that the workshop format worked well, enabling detailed discussion of the QRI diagnosis of recruitment difficulties in relation to RCT teams’ experiences of enrolment and knowledge about their RCT, supplemented by additional short QRI-informed training sessions.

The QRI analysis and workshop as applied in 2018–2019 are thus retained in the finalised QRI-Two (Table 5) which provides the diagnosis of recruitment barriers impacting on the RCT and some potential remedial actions. RCT teams can then use this information to develop and implement their own remedial strategies, apply for an extension, and/or establish further collaboration to improve recruitment and complete enrolment. In some cases, however, the QRI-findings can confirm that the barriers are insurmountable, leading to closure (as in one RCT here). In four of the RCTs, further collaboration with the QuinteT team was commissioned. In two, this involved additional QRI-informed training to assist in the implementation of the team’s own remedial strategies. In two others, the collaboration was more comprehensive and became similar to phase II of the published QRI—requiring time and resources for RCT protocol revisions, data collection, and implementation of QRI-informed training and targeted actions to tackle the newly diagnosed recruitment barriers.

The finalised QRI-Two includes the analysis of RCT documents and accrual data to diagnose the RCT’s specific recruitment barriers and a workshop to include the RCT team’s knowledge and experience and decide the next steps—further work to improve recruitment or informed closure. The further work—remedial actions to improve recruitment—may be undertaken by the RCT team or involve further collaboration. The finalised QRI-Two protocol is presented in Table 5 for future use.

## Discussion

In this study of 32 RCTs across Europe and the USA from 2007 to 2019, a new intervention, the QRI-Two, was developed to provide opportunities to improve recruitment in RCTs underway with enrolment shortfalls. The QRI-Two was shown to be able to identify an array of previously unknown or underestimated and potentially remediable recruitment barriers in all 26 actively recruiting RCTs struggling with enrolment studied here. RCT teams had previously tried their usual improvement strategies, but were still struggling, with some facing closure. The rapid QRI-analysis of existing study documents (RCT protocol, patient information) and screening/accrual data found previously hidden or underestimated challenges linked to the intellectual and emotional demands placed on recruiters and patients by the enrolment process. Evidence of, or clues to, the existence of these hidden challenges were indicated in RCT documents by vague eligibility criteria, inconsistent descriptions of interventions, or mis-interpretable/ambiguous terminology, or in variations in accrual data patterns between sites or over time. Triangulated clues and evidence could be linked to underlying difficulties such as discomfort with equipoise and decisions to exclude some eligible patients. New insights were also provided about other issues, such as why only small numbers of eligible patients were approached in some sites, and how patient preferences could emerge from unclear study presentation. The QRI-Two workshop provided a forum for open discussion of the RCT’s newly diagnosed barriers in the context of the RCT team’s knowledge of the RCT’s design and clinical nuances, with opportunities to consider whether barriers were insurmountable or remedial actions could be developed and implemented to improve recruitment. It was feasible to apply the QRI-Two rapidly and it was confirmed that the intervention was most relevant for actively enrolling RCTs experiencing shortfalls.

Poor RCT recruitment is a common difficulty that besets many RCTs, resulting in delayed and underpowered evidence, and wasted resources. Systematic reviews of the evidence base have identified some simple interventions but only a few with evidence of effectiveness [5–7, 10–13]. QRIs undertaken with RCTs anticipating severe recruitment challenges have exposed the fragile, protracted, and evolving nature of the RCT recruitment process, explaining why a complex intervention with a portfolio of actions is needed to optimise recruitment [17, 18]. The original published QRI, integrated with an RCT from the design stage, offers the prospect of preventing the development of some barriers and addressing recruitment difficulties as they emerge in RCTs anticipating serious challenges [14]. The QRI-Two builds on insights about recruitment derived from completed QRIs. The ethos underlying all QRIs is that it is essential to understand the actual obstacles and challenges hampering an RCT (not just anticipated barriers) so that appropriate data can be collected, issues and discomforts can be discussed, and targeted remedial actions implemented to improve recruitment. This approach is further supported by a systematic review focused on the role of clinicians in RCTs [12] and the findings of a study of 172 RCTs discontinued due to poor recruitment that concluded most had potentially remediable barriers related to issues related to patient eligibility and strong patient and recruiter views about interventions [7].

The QRI-Two offers the opportunity to identify previously hidden but potentially remediable recruitment barriers in RCTs underway with serious enrolment shortfalls. Recruitment barriers found in the actively enrolling RCTs in this analysis included unclear study presentation, difficulties with eligibility and equipoise, losses of eligible patients on the recruitment pathway, strong patient intervention preferences, and a reluctance by recruiters to approach eligible patients. After the diagnosis of recruitment barriers in a QRI-Two, there is then the question of what to do next. The original (integrated) QRI protocol has two phases, with phase I providing an understanding of the recruitment issues (particularly hidden challenges) and phase II comprising the implementation of strategies to prevent or address challenges and optimise recruitment [14]. The finalised QRI-Two protocol is similar in that it diagnoses the barriers and identifies potential remedial strategies to improve recruitment that RCT teams need to implement. In a small number of RCTs, the QRI-Two can find that the recruitment issues are insurmountable (for example because recruiters cannot find a position of equipoise, or following changes in practice), and this may lead to the RCT closing. However, more typically the QRI-Two identifies new or underestimated barriers with clearer insights that enable the development of targeted remedial actions.

In QRIs integrated from the start, analysis of interviews and audio-recordings of recruitment appointments have provided the evidence for the development of supportive training and other actions to address recruitment challenges [15, 20–23, 36, 37]. These remedial actions could also be implemented in RCTs after the QRI-Two. Following the rapid diagnosis of recruitment barriers, time is needed for revision and ethical approval of RCT protocol and/or patient information materials, and then implementation of actions. Such actions need care to tackle such deep-seated barriers and to avoid the development of defensiveness in RCT teams after months or years of shortfalls. Such actions and their evaluation may require additional resources during the remainder of the recruitment period or with extensions. The balance of additional costs needs to be considered in the context of the magnitude of the costs of current problems with enrolment shortfalls leading to delayed evidence, underpowered RCTs, long extensions, and premature closures.

An interesting observation among these RCTs was the limited availability of data about the recruitment process/pathway. Most RCTs were collecting some screening/accrual data, although very few used these data to investigate recruitment barriers, tending to focus on monitoring accrual rates compared with targets and transferring to the data centre only items necessary to comply with the CONSORT guidelines (simply numbers ‘assessed for eligibility’ and ‘excluded’) [38]. These crude criteria on their own reveal little about sources of recruitment difficulties. In a QRI integrated from the start, data are collected about the recruitment process using the SEAR (Screening, Eligibility, Approached, and Randomised) framework [21]. In the QRI-Two workshops, the RCT teams began to appreciate how such data could be used to identify previously hidden recruitment challenges. Some were able to use their existing target-based data in new ways, but many RCT teams and data centres were unsure about how to make protocol modifications or find resources to collect and analyse such data. The lack of data collected about the recruitment process could be a major reason why barriers are so poorly understood. Without a concerted effort, recruitment will continue in many RCTs in a ‘data-free zone’, with little prospect of ever understanding their actual (evidence-based) recruitment challenges.

### Strengths and limitations

An important limitation of this study is the lack of quantitative evaluation of the QRI-Two. This was not funded or included in the commissioning process, where the focus was on identifying recruitment barriers. The lack of randomised evaluation of either the integrated QRI or QRI-Two is also an important limitation. There is observational evidence showing the effectiveness of QRIs improving and completing recruitment in five RCTs (one was a QRI-Two) [24], and QRI-informed training increasing recruiter confidence [37]. In addition, four very difficult RCTs have now recruited, completed follow-up, and published outcomes with the support of a QRI [16, 25–27], and many more have completed recruitment and are in follow-up or continue to accrue. When closure occurs, there is a clear understanding of the insurmountable problems. The QRI is a complex intervention applied prospectively and holistically to optimise recruitment in each RCT, and so a randomised evaluation is difficult to design because of issues of contamination between sites and the reluctance of RCT teams to have control sites. However, the QRI-Two could be evaluated by randomising RCTs underway with enrolment difficulties either to a QRI-Two (preferably with funding for implementation of remedial actions) or continuing as usual, with a primary outcome of numbers randomised or percentage of eligible patients randomised. This paper has provided evidence of the ability of the QRI-Two to identify previously unknown, potentially remediable barriers as well as its feasibility and relevance for actively enrolling RCTs. A randomised evaluation of its impact on recruitment is needed.

Another important limitation was that this analysis was undertaken by members of the QuinteT team, and so lacks independence, although this did enable the inclusion of a wide range of data collected from all 32 RCTs and QRIs. Very few studies of recruitment include data from so many RCTs. Here, data were extracted systematically by the QRI researcher and synthesised by JLD, MJ, and LR. The investigation stretched over 15 years because small numbers of QRIs were undertaken initially in ongoing RCTs. The 20 QRI-Two undertaken 2018–2019 were critical for the finalisation process. It was a strength to include RCTs underway across Europe and the USA, and a variety of RCTs aiming to answer difficult clinical questions in many clinical specialties. The inclusion of RCTs at start-up, without enrolment data, confirmed that the QRI-Two was best applied to RCTs underway with enrolment shortfalls. The findings of this study cannot be generalised to all RCTs, but the commonality of the problems and consistency of findings across so many RCTs suggests wide relevance. An additional strength is that the QRI-Two can be applied rapidly to diagnose recruitment difficulties, as new or identifiable patient data are not needed—important in the context of so many RCTs struggling with recruitment. The finalised QRI-Two protocol is presented in Table 5 for future use. This could be with the QuinteT team or alternatively other research/RCT groups can use it alongside QRI training themes and materials available via publications [20–23, 36, 37], or from existing training courses [35], and there is a future aim to develop ‘train-the-trainer’ programmes.

## Conclusion

The QRI-Two protocol was finalised after development in 12 RCTs and application in 20 RCTs in Europe and the USA. The QRI-Two can be rapidly applied to RCTs underway with serious enrolment shortfalls or threatened with closure to diagnose the array of previously unrecognised or underestimated recruitment challenges hampering enrolment. This provides the opportunity for RCT teams to determine whether the challenges are insurmountable or could be addressed by developing and implementing targeted remedial actions to improve the recruitment process and complete enrolment. The effectiveness of the QRI-Two in improving recruitment requires further evaluation.

## Supplementary Information

**Additional file 1: Table S1.** Summary of findings from QRIs attempted with ongoing RCTs with enrolment shortfalls 2007-2017 (n-12). **Table S2.** Summary of findings from QRI-Two undertaken with actively recruiting RCTs with enrolment shortfalls, 2018-2019 (n-14). **Table S3.** Summary of findings from QRI-Two undertaken with RCTs at the start-up stage, 2018-2019 (n-6).

## Data Availability

The datasets generated for and analysed in the current study are included in this published article and its supplementary tables. Further details about the RCTs or QRIs may be sought from the corresponding author.
