# Supplementary material for: Development of a new adapted QuinteT Recruitment Intervention (QRI-Two) for rapid application to RCTs underway with enrolment shortfalls—to identify previously hidden barriers and improve recruitment
Source: Trials. 2022 Apr 4;23:258. doi: 10.1186/s13063-022-06187-y (PMC8978173; doi:10.1186/s13063-022-06187-y)
Supplement: Supplementary file 1 — Additional file 1: Table S1. Summary of findings from QRIs attempted with ongoing RCTs with enrolment shortfalls 2007-2017 (n-12). Table S2. Summary of findings from QRI-Two undertaken with actively recruiting RCTs with enrolment shortfalls, 2018-2019 (n-14). Table S3. Summary of findings from QRI-Two undertaken with RCTs at the start-up stage, 2018-2019 (n-6). [file 13063_2022_6187_MOESM1_ESM.docx]

**Development of a new adapted Quintet Recruitment Intervention (QRI-Two) for rapid application to RCTs underway with enrolment shortfalls - to identify previously hidden barriers and improve recruitment**

**Supplementary materials**

**Table S1 Summary of findings from QRIs attempted with ongoing RCTs with enrolment shortfalls 2007-2017 (n-12)**

| RCT | Specialty | Design | Compliance with QRI Phase I elements | New initiative | Recruitment difficulties identified | QRI Phase II actions | RCT outcome |
| --- | --- | --- | --- | --- | --- | --- | --- |
| I1 | Oncology | Parallel group 2 arms* (radiation v conservative) | Observation of study meetings only. |  | Equipoise, preferences, study presentation and terminology. | Advice to RCT CI/team. Feedback to study group (n=3), ‘Tips’. | Completed and published [1] |
| I2 | Pediatrics/  primary care | Parallel group 3 arms (drugs) | (ii) pathway mapping (iii) audio-recordings (iv) review documents | None | Eligibility, equipoise, approaching patients. | Report to RCT CI. Group and individual feedback/training. | Completed and published [2] |
| I3 | Oncology/  post-surgery | Parallel group  4 arms* (modes of follow-up) | (i) interviews  (ii) pathway mapping (iii) audio-recordings  (iv) review documents | None | Equipoise, pathways. | Report to RCT CI. Group feedback/training. | Completed and published [3] |
| I4 | Psychiatry | Parallel group 2 arms* (social intervention v control) | (i) interviews  (ii) pathway mapping (iv) review documents | None | Approaching patients, eligibility, equipoise, study presentation and terminology. | Report to RCT CI. Group feedback/training, discussion of eligibility issues, ‘Tips’ | Completed and published [4,5] |
| I5 | Oncology/  Urology surgery | Parallel group 2 arms* (surgery v. radiation) | (i) interviews  (ii) pathway mapping (iii) audio-recordings (iv) review documents | Review recruitment data. | Pathway, eligibility, equipoise, preferences, approaching patients, study presentation and terminology. | Report to CI. Group and individual feedback/training, re-drafted patient information, centre reviews, ‘Tips’. | Closed - poor recruitment. QRI Report published [6] |
| I6 | Vascular surgery | Parallel group 3 arms* (three surgeries) | (ii) pathway mapping  (iii) audio-recordings  (iv) review documents | Review recruitment data. | Study presentation and terminology, pathway issues. | Report to investigator and CI. Individual feedback, ‘Tips’, Q-QAT presentation timing.[7] | Completed and published [8] |
| I7 | Orthopedic surgery | Parallel group 3 arms* (surgery, usual care, placebo) | (i) interviews  (ii) pathway mapping  (iii) audio-recordings  (iv) review documents | Review recruitment data. | Equipoise, study presentation and terminology, approaching patients, recruitment pathway. | Report to RCT CI. Group and individual feedback/training, re-draft patient information, centre reviews, ‘Tips’. | Completed and published [9] |
| I8 | Vascular surgery | Parallel group 2 arms (two surgeries) | (i) interviews  (ii) pathway mapping  (iii) audio-recordings  (iv) review documents | Review recruitment data and observe meetings. | Equipoise, eligibility, study presentation and terminology, preferences, pathways. | Report to RCT CI. Group feedback/training, suggested re-draft of patient information, ‘Tips’. | Recruitment continues |

| RCT | Specialty | Design | Compliance with QRI Phase I elements | New initiative | Recruitment difficulties identified | Phase II QRI actions | RCT outcome | |
| --- | --- | --- | --- | --- | --- | --- | --- | --- |
| I9 | Urology surgery | Parallel group 2 arms (two surgeries) | (i) interviews  (iv) review documents | Review recruitment data.  Workshops. | Equipoise, eligibility, approaching patients, study presentation and terminology. | Report to RCT CI. Feedback and QRI-informed training. | Recruitment completed. |  |
| I10 | Respiratory surgery | Parallel group 2 arms* (surgery v conservative) | (i) interviews  (iv) review documents | Review recruitment data. Workshop. | Equipoise, eligibility, pathways, approaching patients. | Report to RCT CI. Feedback to CI and Trial Manager, future QRI shelved as RCT closed. | Closed due to poor recruitment. | |
| I11 | Vascular surgery | Parallel group 2 arms (two surgeries) | (i) interviews  (iv) review documents | Review recruitment data.  Workshop. | Equipoise, eligibility, pathways, study presentation and terminology. | Report to RCT CI. Feedback and QRI-informed training, revised patient information. | Extended to complete recruitment. | |
| I12 | Ear, nose and throat surgery | Parallel group 2 arms* (surgery v conservative) | (iv) review documents | Review recruitment data.  Workshop | Equipoise, eligibility, study presentation and terminology. | Report to RCT CI. Feedback and QRI-informed training. | Recruitment continues | |

**Feedback received**

I2 “The reflection process, feedback, analysis and discussions during the workshop helped” [2] p.27.

I9: “We successfully completed I9 recruitment on [date] …I think the two QRI meetings were very important in ensuring the success of I9 and we very much appreciated being able to discuss issues with you and the efforts you made. I think the process ought to be used by all trials, either in the setting up period or during the initial months when a trial is getting all the centres up and running.”

**References**

1. Mulvenna P, Nankivell M, Barton R et al. Dexamethasone and supportive care with or without whole brain radiotherapy in treating patients with non-small cell lung cancer with brain metastases unsuitable for resection or stereotactic radiotherapy (QUARTZ): results from a phase 3, non-inferiority, randomised trial. Lancet 2016, 388, 10055: 2004-2014
2. Hay A D, Redmond N M, Costelloe C, Montgomery A A, Fletcher M. Paracetamol and Ibuprofen for the treatment of fever in children: the PITCH randomised controlled trial. Health Technol Assess 2009;13(27)
3. Primrose JN, Perera R, Gray A, Rose P, Fuller A, Corkhill A, et al. Effect of 3 to 5 years of scheduled CEA and CT follow-up to detect recurrence of colorectal cancer: the FACS randomized clinical trial. JAMA 2014; 311:263-70.
4. Howard L, de Salis I, Tomlin Z, Thornicroft G, Donovan JL. Why is recruitment to trials difficult? An investigation into recruitment difficulties in an RCT of supported employment for people with severe mental illness. Contemporary Clinical Trials 2009; 30: 40-46.
5. Heslin M, Howard L, Leese M, et al. Randomized controlled trial of supported employment in England: 2-year follow-up of the Supported Work and Needs (SWAN) study. World Psychiatry. 2011;10(2):132–137.
6. Paramasivan S, Huddart R, Hall E, Lewis R, Birtle A, Donovan JL. Key issues in recruitment to randomized controlled trials with very different interventions: a qualitative investigation of recruitment to the SPARE trial. Trials 2011; 12: 78
7. Paramasivan, S., Strong, S., Wilson, C., Campbell, B., Blazeby, J. & Donovan, J. A simple technique to identify key recruitment issues in randomised controlled trials: Q-QAT - quanti-qualitative appointment timing. Trials. 2015; 16: 88.
8. Brittenden J, Cotton SC, Elders A et al. A Randomized Trial Comparing Treatments for Varicose Veins. N Engl J Med 2014; 371:1218-1227.
9. Beard, D. J., Rees, J. L., Cook, et al. Arthroscopic subacromial decompression for subacromial shoulder pain (CSAW): a multicentre, pragmatic, parallel group, placebo-controlled, three-group, randomised surgical trial. 2018, Lancet: 391 (10118): 329-38.

**Table S2 Summary of findings from QRI-Two undertaken with actively recruiting RCTs with enrolment shortfalls, 2018-2019 (n-14)**

| RCT | Specialty | Design | Rapid analysis and workshop | Recruitment difficulties identified | QRI-informed training ‘taster’ sessions delivered | Collaborative work after workshop |
| --- | --- | --- | --- | --- | --- | --- |
| QT1 | Radiation oncology/  surgery | Parallel group 2 arms  (radiation v surgery | Meeting with CI and coordinator November 2017. QRI presentation November 2018 | Pathway, limited data/analysis, equipoise, eligibility, preferences, and study presentation. | Pathway/SEAR framework. Hidden challenges with eligibility and equipoise. Managing preferences. Study presentation. | Review and revision of screening data, pathway mapping, revision of patient information, QRI-informed training, site review calls and visits. Internal evaluation showed recruitment improved after meeting, workshop, and some site visits/calls. Reached ‘ramp up’ goal earlier than projected. |
| QT2 | Breast surgery | Parallel group 2 arms*  (surgery v conservative) | Meetings with CI and RCT team from June 2018. QRI presentation  November 2019 | Pathway, limited data/analysis, equipoise, preferences, and study presentation. | Managing preferences. Hidden challenges with eligibility and equipoise. Pathway/SEAR framework. Study presentation. | Review and revision of recruitment data, extensive revision of patient information, site reviews and QRI-informed training from Sept 2020 when new materials approved. QRI action implementation delayed by extensive revision of materials and approval processes. QRI-training and site calls now underway. |
| QT3 | Neurology surgery | Parallel group 2 arms*  (surgery v conservative) | QRI analysis and workshop November 2018 | Study presentation, limited data/analysis, equipoise, eligibility, preferences. | Pathway/SEAR framework. Hidden challenges with eligibility and equipoise. Study presentation. | None |
| QT4 | Orthopedic surgery | Parallel group 2 arms*  (surgical v. non-surgical treatment) | QRI analysis and workshop November 2018 | Equipoise, eligibility, limited data/analysis, study presentation. | Pathway/SEAR framework. Hidden challenges with eligibility and equipoise. Study presentation. | None |
| QT5 | Orthopedic surgery | Parallel group 2 arms*  (surgery v conservative) | QRI analysis and workshop November 2018 | Limited data/analysis, study presentation, equipoise, eligibility. | Pathway/SEAR framework. Hidden challenges with eligibility and equipoise. Study presentation. | None |
| QT6 | Gynecology | Parallel group 2 arms*  (intervention v. usual care) | QRI analysis and workshop November 2018 | Study presentation, equipoise, eligibility, financial incentives, limited data/analysis. | Pathway/SEAR framework. Hidden challenges with eligibility and equipoise. Study presentation. | None |

| RCT | Specialty | Design | Rapid analysis and workshop | Recruitment difficulties identified | QRI-informed training ‘taster’ sessions delivered | Collaboration after workshop |
| --- | --- | --- | --- | --- | --- | --- |
| QT7 | Bariatric surgery | Parallel group 2 arms  (two surgeries) | QRI analysis and 2 workshops March 2019 | Limited data/analysis, study presentation, equipoise, eligibility, preferences. | Pathway/SEAR framework. Study presentation. Hidden challenges with eligibility and equipoise. Managing preferences. | Additional training workshop held. Internal survey: workshops rated highly; numbers agreeing to be randomized increased in sites attending workshops compared with non-attenders. |
| QT8 | Head & neck surgery/  radiation | Parallel group 2 arms (surgery v radiation) | QRI analysis and 3 workshops July- September 2019 | Equipoise, eligibility, study presentation and communication issues. | Pathway/SEAR framework. Study presentation. Hidden challenges with eligibility and equipoise. Managing preferences. | Two additional training workshops held. Internal audit of recruitment showed increase in numbers of patients agreeing to be randomized per month. |
| QT9 | Cardio-vascular medicine | Placebo controlled  2 arms (standard drug) | QRI analysis and workshop August 2019 | Pathway, limited data/analysis, equipoise, eligibility, approaching patients, study presentation. | Pathway/SEAR framework. Study presentation. | None |
| QT10 | Cardio-vascular medicine | Parallel group, 2 arms (standard drugs) | QRI analysis and workshop October 2019 | Pathway, limited data/analysis, approaching patients, study presentation | Pathway/SEAR framework. Study presentation. Recruiter role discomfort. | None |
| QT11 | Orthopedic medicine | Placebo controlled 2 arms (standard drugs) | QRI analysis and workshop October 2019 | Pathway, limited data/analysis, eligibility, equipoise, study presentation. | Pathway/SEAR framework. Hidden challenges with eligibility and equipoise. Study presentation. | None |
| QT12 | Cardiac surgery | Parallel group 2 arms*  (surgery v usual care) | QRI analysis and workshop October 2019 | Pathway, limited data/analysis, equipoise, eligibility, study presentation, preferences. | Pathway/SEAR framework. Hidden challenges with eligibility and equipoise. Managing strong patient preferences. | None |
| QT13 | Gastro-enterology | Placebo controlled 3 arms, two drugs | QRI analysis and workshop October 2019 | Pathway, limited data/analysis, approaching patients, equipoise, eligibility, and study presentation. | Pathway/SEAR framework. Hidden challenges with eligibility and equipoise. Recruiter role discomfort. Study presentation. | None |
| QT14 | Psychiatry | Placebo controlled 2 arms (drugs) | QRI analysis and workshop November 2019 | Pathway, limited data/analysis, equipoise, eligibility, study presentation. | Pathway/SEAR framework. Hidden challenges with eligibility and equipoise.  Study presentation. | None |

**Feedback**

QT1: “Very inspiring.” “We have hit our ramp-up goal almost two months earlier than our projected date.” “Your assistance and intervention was very instrumental in helping achieve this goal.”

QT2: “After some choppy waters we are all now resolute that Quintet holds the solutions we need to improve the quality of what we do in RCT.” “I sense we are all struck by the step change for the better in these documents” “This has been a tremendous effort and is an exemplar of what should be provided for every trial.”

QT5: “We had of course already thought through how to streamline the (recruitment) process as best we could, but the way they (QuinteT) did it in a very structured way gave food for thought about how we could improve our own processes.”

QT7: “What I learned is to be more careful in all parts of the process and work on identifying obstacles such as missing recruiting patients that are eligible. We will change the text on our information slides for the study to be more neutral. Trying to make inclusion simple and removing bottlenecks is certainly something to work more with.”

QT8: “Thank you for your great work so far. I found it excellent and very helpful”

**Table S3 Summary of findings from QRI-Two undertaken with RCTs at the start-up stage, 2018-2019 (n-6)**

| RCT | Specialty | Design | Rapid analysis and workshop | Recruitment difficulties identified | QRI-informed training ‘taster’ sessions delivered | Collaborative work after workshop |
| --- | --- | --- | --- | --- | --- | --- |
| QT15 | Pediatrics | Factorial 3 arms* (2 drug v none and conservative | QRI analysis (no recruitment data) and workshop February 2019 | Potential for issues with equipoise, eligibility, study presentation and terminology, and strong patient preferences. | Hidden challenges with eligibility and equipoise. Recruitment pathway and SEAR framework. Study presentation and terminology including randomization. | None. |
| QT16 | Orthopedic medicine | Parallel group 3 arms* (3 conservative) | QRI analysis (no recruitment data) and workshop at start-up meeting August 2019 | Potential for issues with pathway complexity, equipoise, eligibility, study presentation and terminology, and strong patient preferences. | Recruitment pathway and SEAR framework. Study presentation and terminology issues. | None |
| QT17 | Psychiatry | Placebo controlled 3 arms (drugs) | QRI analysis (no recruitment data) and workshop September 2019 | Potential for issues with recruitment pathway complexity and study presentation and terminology issues. | Recruitment pathway and SEAR framework. Study presentation. | None. |
| QT18 | Nephrology | Placebo controlled 2 arms (drugs) | QRI analysis (no recruitment data) and workshop at start-up meet September 2019 | Potential for issues with recruitment pathway complexity, equipoise, eligibility, strong patient preferences, and study presentation and terminology issues. | Hidden challenges with eligibility and equipoise. Recruitment pathway and SEAR framework. Study presentation and terminology (including randomization, placebo, blinding). | None. |
| QT19 | General surgery | Parallel group 3 arms* (three surgeries) | QRI analysis (no recruitment data) and workshop at start-up meet November 2019 | Potential for issues with recruitment pathway complexity, equipoise and eligibility, study presentation and terminology, and strong patient preferences. | Recruitment pathway and SEAR framework. Hidden challenges with eligibility and equipoise. Study presentation and terminology. Managing patient preferences. | None |
| QT20 | Orthopedic medicine | Parallel group 2 arms* (conservative v. physical therapy) | QRI analysis (no recruitment data) and workshop at start-up meet November 2019 | Potential for issues with recruitment pathway complexity, equipoise and eligibility, patient approach (recruiter discomfort), study presentation and terminology, and strong patient preferences. | Recruitment pathway and SEAR framework. Hidden challenges with eligibility and equipoise. Study presentation and terminology. Managing patient preferences. | None |

Feedback

QT15: “We all really enjoyed the training and have been talking a lot about since.”

QT18 “your presentation was thought provoking and idea generating and led to important conversations the next day and the subsequent week. These are exactly the sort of conversations I am happy to see occurring before we begin recruitment and experience the (inevitable) struggles with recruitment and retention. I’m optimistic that having these conversations will help us to be more knowledgeable and effective in communicating our study.”
